# Bioactive Phenolics and Polyphenols: Current Advances and Future Trends

**DOI:** 10.3390/ijms21176142

**Published:** 2020-08-26

**Authors:** Daniel A. Jacobo-Velázquez, Luis Cisneros-Zevallos

**Affiliations:** 1 Tecnologico de Monterrey, Escuela de Ingeniería y Ciencias, Ave. Eugenio Garza Sada 2501, Monterrey NL C.P. 64849, Mexico; 2 Department of Horticultural Sciences, Texas A&M University, College Station, TX 77843-2133, USA

Phenolic compounds are secondary plant metabolites with remarkable health-promoting properties. More than 8000 phenolics have been identified from natural sources [1]. In plants, phenolics play different physiological roles, such as plant growth regulators and as important chemical precursors for the biosynthesis of other molecules such as lignin and suberin, which are produced as a defense mechanism against different biotic and abiotic stresses [2].

Twenty-six contributions (19 research and seven review articles) in this Special Issue show some of the current advances in bioactive phenolics and polyphenols. Research articles published were mainly focused on the evaluation of different bioactivities of phenolics, with an emphasis on the prevention of chronic diseases, whereas another important number of papers described methods for the production of phenolics by chemoenzymatic preparations [3], hairy root culture bioreactors [4], and elicitation of the secondary plant metabolism by preharvest [5] and postharvest [6] abiotic stresses. 

Regarding research published on the effect of phenolics to prevent chronic diseases, it was reported that ellagic acid (a component of ellagitannins, present in crops such as pecans, walnuts, and berries) and its metabolites urolithins A and B, produced by the gut microbiota, differentially regulate fat accumulation and inflammation in 3T3-L1 adipocytes, while not affecting adipogenesis and insulin sensitivity [7]. Another report evaluating the microbial metabolites of chlorogenic acid demonstrated their anti-proliferative effects, S-phase cell-cycle arrest, and apoptosis in human colon cancer Caco-2 cell [8]. Furthermore, carnosol was identified as a component of rosemary extract with potential application as glucose regulating agent by increasing muscle cell glucose uptake via AMPK-dependent GLUT4 glucose transporter translocation [9]. Likewise, the anti-hypertensive effects of polyphenols from acacia (an evergreen tree belonging to the genus *Acacia* in the legume family) was reported, using spontaneously hypertensive rats as an experimental model [10]. Other published papers in this Special Issue showed the in vivo attenuation effect of ischemic myocardial damage by phenolic extracts from *Crataegus oxyacanth* and *Rosmarinus officinalis* [11], whereas it was reported that extracts from *Aspidosperma pyrifolium* presented in vivo anti-inflammatory properties in mice with peritonitis [12]. 

Furthermore, other contributors to this Special Issue reported the potential use of 3,5,6,7,8,3′,4′-heptamethoxyflavone, a *Citrus* flavonoid, for the preparation of skincare products due to its capacity to inhibit collagenase activity and to induce type-I procollagen synthesis in UV-induced human dermal fibroblast neonatal (HDFn) cells [13]. Other bioactive properties of phenolics reported were the inhibition of Zika virus infection by isoquercitin [14], the neuroprotective effects of anthocyanin-enriched extracts from berries [15] and quercetin [16], and the antibacterial effects against *Staphylococcus aureus* of flavonoids from the traditional Japanese medicine keigairengyoto [17]. Isorhamnetin and quercetin derivatives were reported as the anti-acetylcholinesterase principles of marigold (*Calendula officinalis*) flowers and preparations [18], whereas the antinociceptive effect of *Arrabidaea brachypoda* (DC) bureau phenolic extract was also demonstrated [19]. The intestinal permeability, cellular antioxidant activity, and plasma stability of phenolic compounds from mango [20] and isorhamnetin glycoside from *Opuntia ficus-indica* (L.) [21] were also reported in the Special Issue.

The Special Issue included seven review papers that describe the current status of phenolic compounds, covering general aspects of their bioactivity for the suppression of chronic diseases [1], as well as the bioactivity of specific group of phenolics such as the stilbenoids [22,23], ellagitannins, and anthocyanins [24]. In addition, the health-promoting properties of phenolics present in lentils [25] and dry common beans [26] were also reviewed. Finally, a review article describing nanofiltration and tight ultrafiltration membrane techniques for the recovery of polyphenols from agro-food by-products was also presented [27]. 

The evaluation of phenolics bioactivities from different plant sources is a growing area of research. Every year, new scientific information supports the increased potential of phenolics to prevent different chronic and degenerative diseases. Further research should continue the direction of identifying natural sources rich in phenolics and evaluating their bioactivities. However, now that the bioactivity of phenolics from several plant materials has been characterized, further research efforts could be focused on taking the generated fundamental science into the market through developing innovative food products and dietary supplement formulations. One interesting emerging area of research is the design of effective nutraceutical combinations in the form of foods, beverages, and dietary supplements that could be used not only in the prevention of chronic disease but also for their treatment [28]. In this context, the synergistic combination of phenolic compounds with other nutraceuticals should be evaluated for the prevention and treatment of chronic diseases [29]. Furthermore, the development of bioprocesses to obtain next-generation functional food and beverages is crucial to reach the market and provide the desired beneficial effect of phenolic compounds to the population. In this context, it has been recently proposed that the application of postharvest abiotic stresses in horticultural crops to increase the content of phenolic compounds, and their further transformation into processed food products using nonthermal processing technologies, could be an effective approach to obtain shelf-stable products with a high content of antioxidant phenolic compounds [30]. 
